# Investigation of Pharmacokinetic Parameters of Trelagliptin in Egyptian Volunteers Using Sensitive LC-MS/MS: A Comparative Study with a Japanese Population

**DOI:** 10.1155/2021/9664099

**Published:** 2021-12-09

**Authors:** Shereen Mowaka, Nermeen Ashoush, Mariam M. Tadros, Bassam M. Ayoub

**Affiliations:** ^1^Pharmaceutical Chemistry Department, Faculty of Pharmacy, The British University in Egypt, El-Sherouk City, Cairo, Egypt; ^2^The Center for Drug Research and Development (CDRD), Faculty of Pharmacy, The British University in Egypt, El-Sherouk City, Cairo, Egypt; ^3^Analytical Chemistry Department, Faculty of Pharmacy, Helwan University, Ain Helwan, Cairo, Egypt; ^4^Clinical Pharmacy Practice Department, School of Pharmacy, NewGiza University, Cairo, Egypt; ^5^Pharmaceutical Analytical Chemistry Department, Faculty of Pharmacy, Ain Shams University, El-Abaseya, Cairo, Egypt

## Abstract

Trelagliptin (TLN) is a novel once-weekly antidiabetic drug that enhanced the patient compliance in type 2 diabetes. TLN analysis and bioanalysis literature review showed many methods for TLN assay either in dosage form or as biological fluids (pharmacokinetic parameters), but all those methods did not consider the full details dealing with biological assay of TLN. Studies that included information about pharmacokinetic parameters did not mention the used analytical procedures for those determinations and parameters. Although some LC-MS/MS and UPLC-UV methods were reported for TLN bioassay in rats' plasma, they used direct precipitation techniques, and the current described procedure showed lower LLOQ than all the reported methods in spite of that working on human plasma is more complicated than on rats' plasma. In this study, LC-MS/MS bioanalysis of TLN in human plasma (4–1000 nM) was employed successfully with LLOQ of 4 nM which is lower than all reported methods in rats' plasma followed by a preliminary pharmacokinetic study. Alogliptin was used as internal standard (IS) because of its structure similarity to TLN. Pharmacokinetic parameters of TLN were investigated in Egyptian volunteers, and they had been compared to Japanese. Liquid-liquid extraction showed more sensitive results than direct precipitation. The proposed method was successfully applied to a pharmacokinetic study conducted on Egyptian volunteers. No dose modification is required upon comparing the pharmacokinetic parameters of the current study and previous studies on non-Egyptian volunteers.

## 1. Introduction

**Figure 1:**
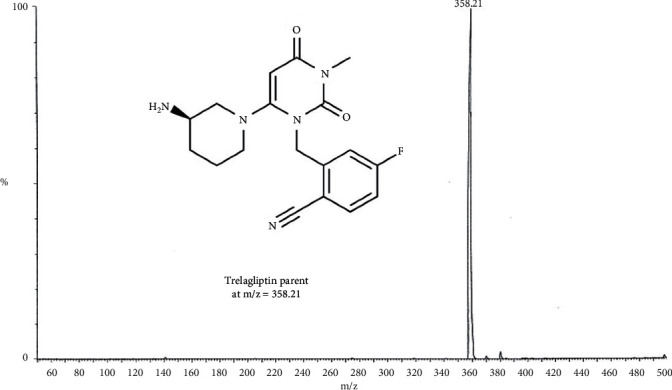
MS of TLN and its chemical structure.

Trelagliptin (TLN, Figure 1) inhibits dipeptidyl peptidase-4 enzyme increasing GLP-1 to treat type 2 diabetes. In addition to its insulin secretagogue effect, it also improves insulin resistance [1]. It had been approved for use in Japan in March 2015 by Takeda pharmaceutical Company as Zafatek^®^ tablets. As a once-weekly drug, it enhances the patient adherence to the treatment regimen instead of the other previously approved gliptins. TLN showed high safety profile with patients suffering from end-stage renal disease or even with renal impairment [2]. Moreover, TLN was repositioned as a potential therapeutic agent for metabolic syndrome with polypharmacologic effects that will lower the treatment cost as one drug with multifaceted therapy [3]. Also, repositioning of TLN and its sister gliptins for neurodegenerative diseases is suggested based on improving insulin resistance in the brain [4]. TLN is a well-tolerated drug with less dosing frequency and less-serious adverse events [5]. TLN clinical trials have confirmed that it can effectively control the plasma concentration of glucose and HbA1c in type 2 diabetic patients [6].

TLN analysis and bioanalysis literature review showed many methods [7–13] for TLN assay either in dosage form or as biological fluids (pharmacokinetic parameters). An LC method for determination of enantiomeric purity of TLN was reported [7]. Some other stability-indicating LC methods were developed for TLN assay in the presence of impurities and/or degradation products [8–11], but all those methods were not related to biological assay of TLN. Studies that included information about pharmacokinetic parameters [5, 12, 13] did not mention the used analytical procedures for those determinations and parameters. Although some LC-MS/MS and UPLC-UV methods were used for TLN bioassay in rats' plasma [14–18], they used direct precipitation techniques, and the current described procedure showed lower LLOQ than all the reported methods in spite of that working on human plasma is more complicated than on rats' plasma. The use of two extracting solvents' mixture (TBME and DEE) besides the upgrading of the detector, to be mass spectrometry instead of photodiode array [15], has greatly encouraged the authors to go further within this preliminary pharmacokinetic study.

In this study, LC-MS/MS bioanalysis of TLN in human plasma (4–1000 nM) was employed successfully with LLOQ of 4 nM which is lower than all reported methods in rats' plasma followed by a preliminary pharmacokinetic study. Pharmacokinetic parameters of TLN were investigated in Egyptian volunteers, and they had been compared to Japanese race results obtained from the literature. Extraction of TLN from plasma enhanced using liquid-liquid extraction followed by vacuum evaporation that showed more sensitive results than direct precipitation.

## 2. Methods

### 2.1. Chemicals and Chromatographic Conditions

TLN (99.0%), Alogliptin as IS (99.2%), ZAFATEK® (50 mg) tablets, TBME, and DEE were thankfully contributed by the Center for Drug Research and Development, at the British University in Egypt, BUE (Cairo, Egypt). HPLC-grade methanol and acetonitrile were purchased from (Sigma, USA). The Phenomenex C_18_ column (1.6 *μ*m, 150 × 2.1 mm), Waters® UPLC-TQ, and Mass *Lynx* software, with a flow rate of 0.3 mL/min, mixture of acetonitrile/0.3 formic acid (90 : 10, *v/v*) as an isocratic mobile phase, and injection V of 10 *μ*L, were used. Cone voltage values of 25 V and 30 V and collision energy values of 60 eV and 55 eV were applied for TLN and IS (Alogliptin), respectively. MRM of m/z 358.2 to 133.9 for TLN and m/z 340.2 to 116.0 for IS in the ESI positive mode were applied.

### 2.2. Calibrators, QC Samples, and Sample Preparation

10 *μ*L from each of the TLN working solutions (0.4, 1.2, 2.5, 10, 40, 50, 70, 80, and 100 *μ*M) had been added to 990 *μ*L blank plasma, so the final concentrations were 4 nM (LLOQ), 12 nM (LQC) 25, 100, 400, and 500 nM (MQC), and 700 and 800 (HQC) and 1000 nM. For the sample preparation, 100 *μ*L of IS (300 nM, solvent as acetonitrile) was added to 250 *μ*L of each sample, and then, 1.5 mL of (TBME and DEE mixture, 50 : 50, *v/v*) was added followed by 15 minutes centrifugation at 15.000 rpm. Withdrawal of clear 1.3 mL from the upper layer was successful, and it was subjected to vacuum and evaporated till dryness and reconstituted with 250 *μ*L methanol before the chromatographic run.

### 2.3. Bioanalytical Validation and Biological Samples

As per FDA bioanalytical validation guidance [19], six different concentrations had been used for the calibration curve estimation and six batches from different plasma sources were checked for selectivity. Both accuracy and precision parameters (*n* = 5) had been evaluated using the calibration parameters (bias, S.D., % RSD) based on bioanalysis of LLOQ, LQC, MQC, and HQC levels. Carry over, matrix factor, and extraction recoveries were evaluated as per FDA bioanalytical validation guidance [19]. Four types of stability were checked for LQC and HQC samples that included leaving the samples for three hours either at room temperature or in the auto sampler, 3 cycles freeze and thaw stability, and 2 weeks (−80 °C) stability.

The pharmacokinetic parameters of TLN were studied in healthy human subjects according to the relevant ethical guidelines and regulations of the World Medical Association Declaration of Helsinki (October 1996) and the International Conference of Harmonization Tripartite Guideline for Good Clinical Practice. Each volunteer before enrollment provided written informed consent. Approval of the study by the ethical committee was mandatory according to the Egyptian Ministry of Health and the British University in Egypt research ethics guidelines. The randomized clinical trial experimental protocol was finally approved by the British University in Egypt (BUE) Faculty of Pharmacy ethical committee, Code : CL/2004, on 05/08/2020 after preliminary discussion and proposal submission in April 2020. The mentioned BUE ethical committee is recognized by the ENREC (Egyptian Network of Research Ethics Committees), http://www.enrec.org/directory. The clinical trial protocol was previously registered in a publically accessible primary register that participates in the WHO International Clinical Trial Registry Platform (ClinicalTrials.gov, registration date: 05/05/2020, ID: NCT04374864), and it is available online at (https://clinicaltrials.gov/ct2/show/NCT04374864). 1 mL blood samples from six human volunteers (25–39 years) was collected at 0, 0.5, 1, 1.5, 2, 2.5, 3, 8, 24, 48, 72, 96, 120, 144, and 168 hrs after oral administration of 50 mg TLN as Zafatek^®^ tablet. The samples were collected in EDTA tubes and centrifuged for 5 min (3000 rpm) then the plasma samples were treated as under sample preparation to calculate the determinations. *C*_max_, *T*_max_, *t*_1/2 (0-96)_, elimination rate constant, AUC_0-t (0-96)_, and AUC_0-inf_ were estimated using a validated excel sheet.

## 3. Results and Discussion

Trelagliptin (TLN) is a novel once-weekly antidiabetic drug that enhanced the patient compliance in type 2 diabetes [20–24]. TLN analysis and bioanalysis literature review showed many methods for TLN assay either in dosage form or as biological fluids (pharmacokinetic parameters), but all those methods did not consider the full details dealing with biological assay of TLN. Studies that included information about pharmacokinetic parameters did not mention the used analytical procedures for those determinations and parameters. Based on previous experience of the authors with the handling of TLN plasma samples either dealing with rats' plasma or human plasma [15], one of the main targets in this study is to enhance TLN extraction that was achieved by liquid-liquid extraction using a mixture of two organic solvents (TBME and DEE). In comparison with previous work where only diethyl ether was used as the extracting solvent for TLN and IS, higher sensitivity was achieved in this presented study where a mixture of two organic solvents was used in addition to acetonitrile added to extract the IS. The use of two extracting solvents' mixture (TBME and DEE) besides the upgrading of the detector, to be mass spectrometry instead of photodiode array, has greatly encouraged the authors to go further into this preliminary pharmacokinetic study.

**Figure 2:**
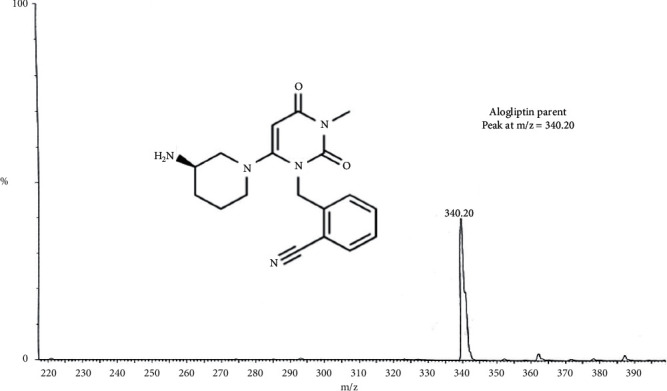
MS of IS and its chemical structure.

**Figure 3:**
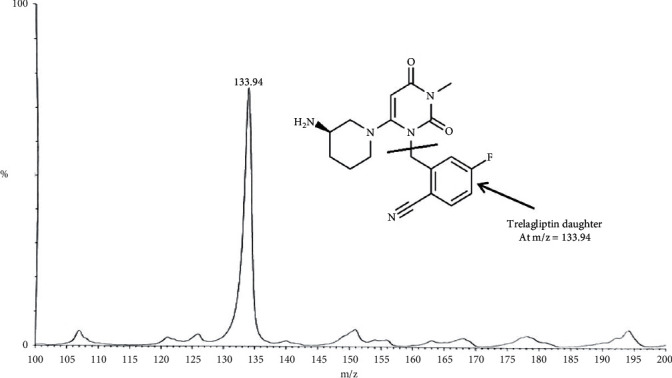
TLN daughter ions (+ESI) at m/z = 133.94.

**Figure 4:**
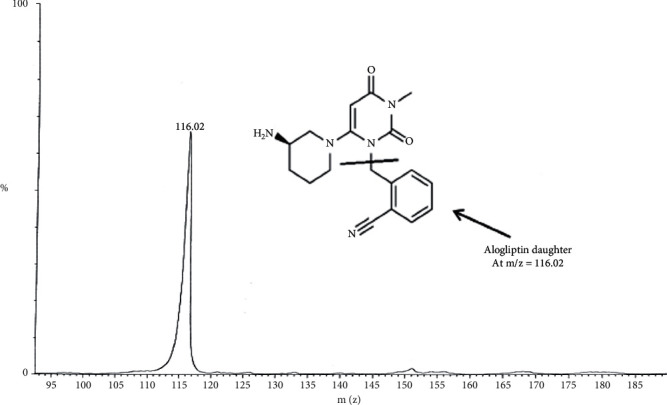
IS daughter ions (+ESI) at m/z = 116.02.

**Figure 5:**
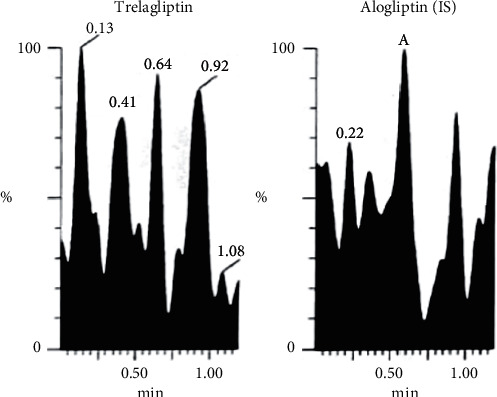
Blank plasma sample (for testing selectivity) showing no interference from endogenous plasma components either with TLN or with IS.

**Figure 6:**
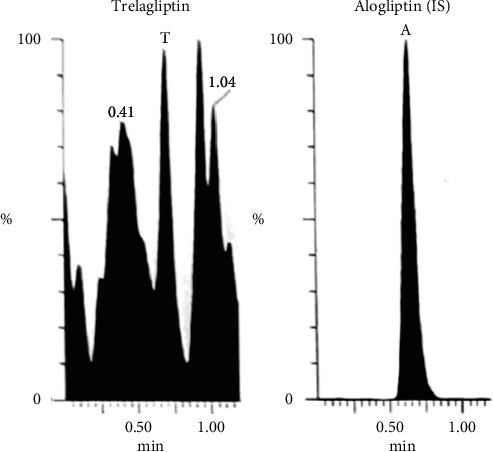
Zero human plasma sample showing IS only.

**Figure 7:**
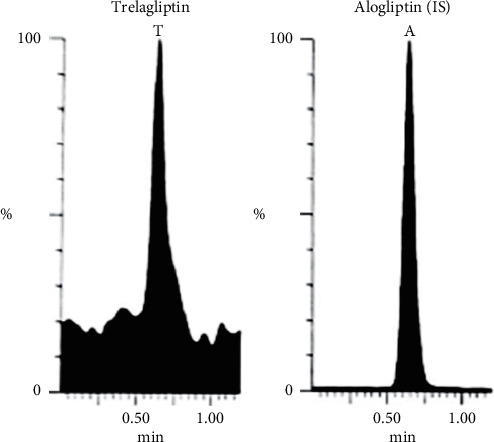
LLOQ sample (4 nMol/L of TLN) using MRM, (m/z = 358.2 to 133.9) and (IS, m/z = 340.2 to 116.0).

**Figure 8:**
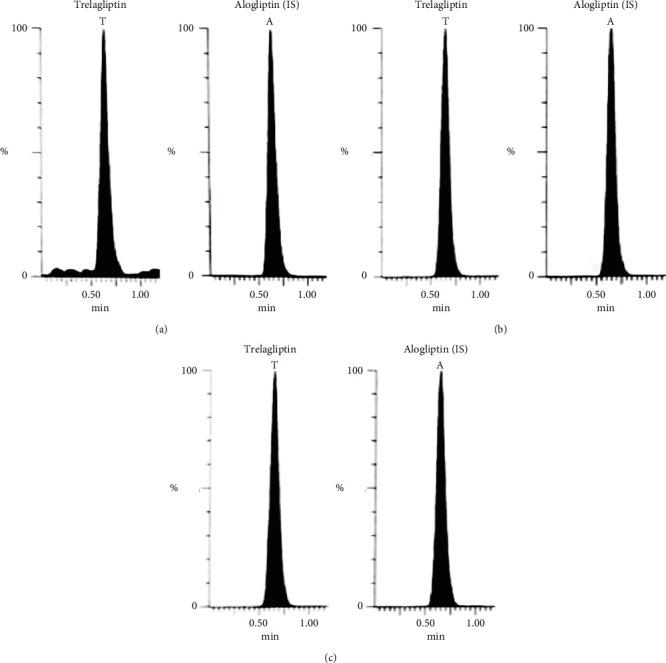
MRM of TLN (m/z = 358.2 to 133.9) and (IS, m/z = 340.2 to 116.0) in LQC, 12 ng/mL (a), MQC, 500 ng/mL (b), and 800 ng/mL HQC (c) spiked human plasma validation samples.

Bioassay (LC-MS/MS) of TLN (4–1000 nM, *y* = 0.0036*x* + 0.0099, *r* = 0.9994) was employed. Positive ESI Multiple Reaction Monitoring of m/z 358.2 to 133.9 for TLN and m/z 340.2 to 116.0 for IS was adopted, as depicted in Figures 1–4. Satisfactory results for selectivity from blank plasma samples without interference, zero sample, and LLOQ sample of 4 nM are shown in Figures 5–7, and all QC samples (Figure 8) are presented. Accuracy and precision showed satisfactory results of ±20% (Table 1). Extraction recovery ranged from 82.92% to 83.85%. Matrix factor ranged from 87.23% to 97.17%. All stability determinations showed recoveries more than 85% (ranged from 89.33% to 96.87%). No carry over was observed after injection of blank after the HQC samples. Dilution integrity samples showed a recovery of 96.9% after dilution 5-folds.

**Figure 9:**
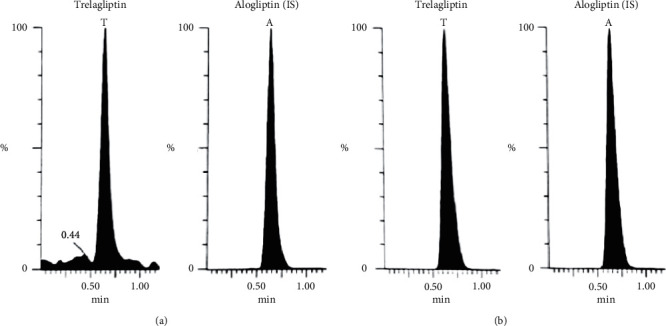
MRM of TLN (m/z = 358.2 to 133.9) and (IS, m/z = 340.2 to 116.0) in the human volunteer plasma sample obtained after (a) 5 days (close to *C*_min_) and (b) after 1 hr (close to *C*_max_) of TLN (50 mg).

**Figure 10:**
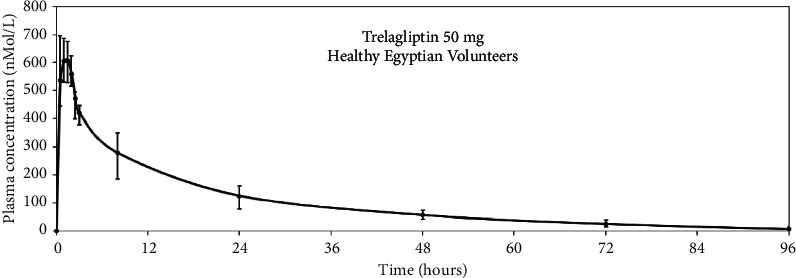
The plasma concentrations versus time curve of TLN after administration of Zafatek® tablet (50 mg TLN).

Successful application of the developed method to a pharmacokinetic study conducted on Egyptian volunteers was employed (Figure 9). Plotting of the mean human plasma concentrations against time is shown in Figure 10. Regarding ethnic difference, the Egyptian pharmacokinetic parameters were compared to Japanese, as previously reported [5, 12, 13]. The calculated pharmacokinetic parameters in the current work were closely related to previous studies conducted in Japanese subjects using 50 mg TLN. The values of *C*_max_, *T*_max_, and AUC_0-∞_ (Table 2) were similar to those data obtained from Japanese [5, 12, 13], while *t*_1/2_ showed some deviation than some studies, but it was close to one study that considered hepatic and nonhepatic impaired patients as 22.6 ± 9.14 [12]. This insignificant difference recommends that no dose adjustment is required in the administration of 50 mg TLN by the Egyptian population. The conducted study was capable of calculating the main parameters although it was not possible to quantify TLN in samples collected between 120 and 168 h due to their low concentrations below the LLOQ of the developed method (4 nM).

## 4. Conclusions

We can conclude that the proposed bioanalytical LC-MS/MS method for TLN using a mixture of two organic solvents was able to estimate TLN in plasma samples with high sensitivity with successful application to a pharmacokinetic study conducted on Egyptian volunteers. No dose modification is required upon comparing the pharmacokinetic parameters of the current study and previous studies conducted on non-Egyptian volunteers.

## Figures and Tables

**Table 1:** Accuracy and precision results for TLN bioassay.

Accuracy and precision (*n* = 5, three days)		LLOQ (4 nMol/L)	LQC (12 nMol/L)	MQC (500 nMol/L)	HQC (800 nMol/L)
*1st day, intraday*	Average recovery	80.0	109.89	92.85	96.28
	Bias (mean, *n* = 5)	−20.0	9.89	−7.15	−3.72
	S.D.	13.71	7.65	4.58	4.05
	% R.S.D	17.14	6.96	4.92	4.21

*2nd day, intraday*	Average recovery	80.76	103.61	99.80	99.40
	Bias (mean, *n* = 5)	−19.24	3.61	−0.20	−0.60
	S.D.	11.09	3.85	2.16	1.03
	% R.S.D	13.74	3.72	2.16	1.04

*3rd day, intraday*	Average recovery	81.32	102.79	97.74	97.20
	Bias (mean, *n* = 5)	−18.68	2.79	−2.26	−2.80
	S.D.	9.80	5.57	2.17	3.10
	% R.S.D	12.05	5.42	2.22	3.19

*Interday*	Average recovery	80.56	105.56	96.80	97.63
	Bias (mean, *n* = 5)	−19.44	5.56	−3.20	−2.37
	S.D.	12.38	6.96	4.31	3.28
	% R.S.D	15.36	6.59	4.45	3.36

**Table 2:** Pharmacokinetic parameters of 50 mg TLN after administration to healthy Egyptian volunteers (*n* = 6).

Pharmacokinetic parameters	Trelagliptin
*C*_max_ (nMol/L) as mean ± S.D. *C*_max_ (highest value–lowest value)	629.23 ± 57.04 (695.47–560.63)
*C*_max_ (ng/mL) as mean ± S.D. *C*_max_ (highest value–lowest value)	225.37 ± 20.43 (249.09–200.80)
*T*_max_ (hours) as mean ± S.D. *T*_max_ (highest value–lowest value)	1.1 ± 0.34 (1.5–0.5)
Elimination rate constant (h^−1^) as mean ± S.D. (highest value–lowest value)	0.046353 ± 0.004213 (0.0498422 and 0.0375328)
*t*_1/2__(0-96)_ (hours) as mean ± S.D. (highest value–lowest value)	15.1 ± 1.6 (18.5 and 14.0)
AUC _0-t_ (nMol^*∗*^h/L) as mean ± S.D. (*t* = 96)	9827.2 ± 1075.8
AUC _0-inf_ (nMol^*∗*^h/L) as mean ± S.D.	9982.5 ± 1027.4
AUC _0-t_ (ng^*∗*^h/mL) as mean ± S.D. (*t* = 96)	3519.8 ± 385.3
AUC _0-inf_ (ng^*∗*^h/mL) as mean ± S.D.	3575.4 ± 368.0

## Data Availability

The data (including figures) used to support the findings of this study are included within the article.

## Additional Points

Samples of Trelagliptin are available from the CDRD Research Center.

## References

[1] Liu Z., Xu L., Xing M. (2020). Trelagliptin succinate: DPP-4 inhibitor to improve insulin resistance in adipocytes. *Biomedicine & Pharmacotherapy*.

[2] Kaku K., Ishida K., Shimizu K., Achira M., Umeda Y. (2020). Efficacy and safety of trelagliptin in Japanese patients with type 2 diabetes with severe renal impairment or end‐stage renal disease: results from a randomized, phase 3 study. *Journal of Diabetes Investigation*.

[3] Rameshrad M., Razavi B. M., Ferns G. A. A., Hosseinzadeh H. (2019). Pharmacology of dipeptidyl peptidase-4 inhibitors and its use in the management of metabolic syndrome: a comprehensive review on drug repositioning. *DARU Journal of Pharmaceutical Sciences*.

[4] Mousa S., Ayoub B. (2019). Repositioning of dipeptidyl peptidase-4 inhibitors and glucagon like peptide-1 agonists as potential neuroprotective agents. *Neural Regeneration Research*.

[5] Kaku K. (2017). Safety evaluation of trelagliptin in the treatment of Japanese type 2 diabetes mellitus patients. *Expert Opinion on Drug Safety*.

[6] Yang J., Zhan S. P., Zhang H. Y. (2017). Clinical application review of the novel once-weekly DPP-4 inhibitor trelagliptin in the treatment of type 2 diabetes. *Chinese Journal of New Drugs*.

[7] Wang Q., Chen X., Zhang C. (2015). Determination of the enantiomeric purity of trelagliptin by pre-column derivatization and liquid chromatography on a chiral stationary phase. *Chromatographia*.

[8] Luo Z., Chen X., Wang G. (2018). Development of a validated HPLC method for the quantitative determination of trelagliptin succinate and its related substances in pharmaceutical dosage forms. *European Journal of Pharmaceutical Sciences*.

[9] Zaghary W. A., Mowaka S., Hassan M. A., Ayoub B. M. (2017). Suitability of various chromatographic and spectroscopic techniques for analysis and kinetic degradation study of trelagliptin. *Scientific Reports*.

[10] Zhang H., Sun L., Zou L. (2016). Identification, characterization and HPLC quantification of process-related impurities in Trelagliptin succinate bulk drug: six identified as new compounds. *Journal of Pharmaceutical and Biomedical Analysis*.

[11] Deng S. S., Li Z. Y., Jiang J. Y. (2016). Determination of related substances in trelagliptin succinate by RP-HPLC and identification of impurities from acid degradation by LC-MS/MS. *Chin. J. New Drugs*.

[12] (2015). Report on the deliberation results. https://www.pmda.go.jp/files/000213963.pdf.

[13] *Company Core Data Sheet 17102017 (CCDS) Zafatek, V.1.1*.

[14] Han Y., Chen L., Liu W. (2019). An HPLC-MS/MS method for quantitation of trelagliptin and application in a comparative pharmacokinetic study. *Bioanalysis*.

[15] Attallah M. A., Mowaka S., Elkady E. F., Fouad M., Ayoub B. (2019). Analysis and bio-analysis of omarigliptin, trelagliptin and alogliptin: applied to biological samples and degradation kinetic study. *Microchemical Journal*.

[16] Ayoub B. M., Mowaka S., Safar M. M. (2018). Repositioning of omarigliptin as a once-weekly intranasal anti-parkinsonian agent. *Scientific Reports*.

[17] Hu X.-x., Lan T., Chen Z. (2016). A rapid and sensitive UHPLC-MS/MS assay for the determination of trelagliptin in rat plasma and its application to a pharmacokinetic study. *Journal of Chromatography B*.

[18] Zhou L., Xi W., Zhang H., Sun L., Yu J., Zou Q. (2019). The chiral bioconversion and pharmacokinetic analysis of trelagliptin in beagle dog plasma by LC-MS/MS. *Journal of Chromatographic Science*.

[19] Food and Drug Administration of the United States (FDA) (2016). *Guidance for Industry: Bioanalytical Method Validation, US Departmentof Health and Human Services*.

[20] Kaku K. (2015). First novel once-weekly DPP-4 inhibitor, trelagliptin, for the treatment of type 2 diabetes mellitus. *Expert Opinion on Pharmacotherapy*.

[21] Grimshaw C. E., Jennings A., Kamran R. (2016). Trelagliptin (syr-472, zafatek), novel once-weekly treatment for type 2 diabetes, inhibits dipeptidyl peptidase-4 (dpp-4) via a non-covalent mechanism. *PLoS One*.

[22] Davis T. M. E. Dipeptidyl peptidase-4 inhibitors: pharmacokinetics, efficacy, tolerability and safety in renal impairment (2014) Diabetes. *Obesity and Metabolism*.

[23] Stoimenis D., Karagiannis T., Katsoula A. (2017). Once-weekly dipeptidyl peptidase-4 inhibitors for type 2 diabetes: a systematic review and meta-analysis. *Expert Opinion on Pharmacotherapy*.

[24] Carnovale C., Mazhar F., Arzenton E. (2019). Bullous pemphigoid induced by dipeptidyl peptidase-4 (DPP-4) inhibitors: a pharmacovigilance-pharmacodynamic/pharmacokinetic assessment through an analysis of the vigibase®. *Expert Opinion on Drug Safety*.

